# Anesthetics as an Alternative to Opioids in Laparoscopic Surgeries: An Observational Study

**DOI:** 10.7759/cureus.86421

**Published:** 2025-06-20

**Authors:** Niyati Pandya, Neeta Patel, Charmi Bhimbha, Nandan Upadhyay, Nishit Mehta, Parth Jani

**Affiliations:** 1 Anaesthesiology, All India Institute of Medical Sciences, Rajkot, Rajkot, IND; 2 Anaesthesiology, C U Shah Medical College and Hospital, Surendranagar, IND; 3 General Practice, All India Institute of Medical Sciences, Rajkot, Rajkot, IND; 4 Internal Medicine, All India Institute of Medical Sciences, Rajkot, Rajkot, IND

**Keywords:** dexmedetomidine, epidural analgesia, intravenous lignocaine, laparoscopic surgery, multimodal analgesia, opioid-free anesthesia, ropivacaine

## Abstract

Background: Laparoscopic surgery, though minimally invasive, often results in significant postoperative pain, particularly in the initial hours. While opioids remain a conventional analgesic choice, their use is associated with adverse effects, including respiratory depression, sedation, nausea, and dependency. This study aimed to evaluate the efficacy of opioid-free multimodal analgesia using epidural with general anesthesia to minimize opioid requirements and related complications.

Methods: This observational study was conducted on 50 patients aged >20 years, with American Society of Anesthesiologists (ASA) grade I, II, and III, posted for surgeries under general anesthesia at C.U. Shah Medical College and Hospital, Surendranagar from January 2021 to June 2022 to evaluate opioid free anesthesia in major laparoscopic surgeries on perioperative hemodynamics and postoperative analgesia. The patients were given general anesthesia with epidural anesthesia.

Results: The study population comprised 42 (84%) female and eight (16%) male patients. Hemodynamic monitoring showed stable parameters with no significant variations in heart rate after non-depolarizing muscle relaxant administration, at one hour, two hours, and four hours postoperatively. Similarly, systolic blood pressure, diastolic blood pressure, and mean arterial pressure remained stable at all measured time points.

Pain assessment using the visual analog scale revealed mild discomfort at extubation (mean score: 2.76 ± 0.64), which progressively decreased over the subsequent 24 hours. Sedation level, measured by the Ramsay Sedation Scale, was at its peak at extubation (3.72 ± 0.48) and gradually declined to near-baseline levels by four hours post extubation (1.96 ± 1.13).

Cognitive function evaluation using the Montreal Cognitive Assessment showed no significant difference between preoperative (27.9 ± 1.37) and 24-hour postoperative (26.72 ± 1.37) scores. The incidence of postoperative nausea and vomiting was 18%, successfully managed with 0.15 mg/kg ondansetron, with no other complications reported.

Conclusion: Opioid-free anesthesia combining epidural ropivacaine with general anesthesia provides effective analgesia in laparoscopic surgeries, as evidenced by stable hemodynamics, acceptable pain scores, minimal sedation, high patient satisfaction, and no significant cognitive impairment. This approach may serve as a viable alternative to opioid-based analgesia, reducing associated side effects.

## Introduction

In balanced general anesthesia, regional anesthesia (e.g., central neuraxial blockade and peripheral nerve blocks) is used alongside concurrently administered systemic agents such as sedatives (e.g., propofol and ketamine), opioids, inhalational agents, muscle relaxants, and stress-reducing medications, aiming for optimal anesthetic effects with minimal complications [1].

Laparoscopic surgery, a minimally invasive technique, offers advantages such as reduced pain, faster recovery, and shorter hospital stays. However, patients often experience significant pain in the first four hours postoperatively due to peritoneal irritation from CO₂ insufflation, bowel manipulation, or retained blood. Effective management of acute postsurgical pain is essential to prevent the development of chronic postoperative pain [2,3].

Opioids remain the cornerstone of perioperative analgesia, providing effective pain relief and hemodynamic stability. Opioids produce their primary effects by binding to specific G-protein-coupled receptors (µ, δ, κ) located on neurons in both the central and peripheral nervous systems. Upon binding, they activate inhibitory G-proteins, triggering intracellular changes: opening potassium channels causes neuronal hyperpolarization (reducing excitability), closing voltage-gated calcium channels inhibits the release of excitatory neurotransmitters (like glutamate and substance P) from presynaptic terminals, and inhibiting adenylyl cyclase lowers cAMP levels. This suppresses pain transmission at multiple levels - presynaptically and postsynaptically in the spinal cord dorsal horn, via descending inhibitory pathways from the brainstem, and by altering pain perception in higher brain centers (thalamus, cortex, limbic system). Simultaneously, opioids blunt the sympathetic nervous system's stress response to surgical stimuli, promoting hemodynamic stability by preventing tachycardia and hypertension, without significant direct myocardial depression at typical doses. Additionally, peripheral receptors on sensory nerves, especially in inflamed tissues, contribute locally to analgesia [4]. However, their use is limited by side effects such as nausea, vomiting, sedation, respiratory depression, tolerance, dependence, constipation, and cognitive impairment. Rare complications include confusion, hallucinations, delirium, and immunosuppression. These adverse effects can prolong recovery, increase hospital stays, and burden healthcare resources [4,5].

The late 18th and early 19th centuries saw a surge in opioid prescriptions, leading to a global opioid epidemic marked by addiction, overdose deaths, and significant public health challenges like unpredictable and potent illicit drug supply, now dominated by fentanyl, which drives an escalating overdose crisis further complicated by widespread polysubstance use. Significant barriers severely limit access to life-saving treatments like medication for opioid use disorder (MOUD) and harm reduction tools, stemming from pervasive stigma, insufficient treatment capacity, insurance hurdles, and regulatory burdens. Furthermore, deep-seated structural disparities and social determinants of health disproportionately harm marginalized communities, worsening addiction outcomes and obstructing recovery pathways. This crisis prompted the medical community to explore opioid-sparing or opioid-free strategies for pain management [6].

Certain patient populations, such as those with obesity, sleep apnea, chronic obstructive pulmonary disease (COPD), complex regional pain syndrome, opioid addiction, or cancer [6,7], are at higher risk of opioid-related complications, making opioid-free anesthesia (OFA) a preferable alternative [8]. OFA employs multimodal analgesia [9,10] using α-2 agonists (e.g., dexmedetomidine), local anesthetics (e.g., lidocaine), nonsteroidal anti-inflammatory drugs (NSAIDs), N-methyl-D-aspartate (NMDA) antagonists (e.g., ketamine), and steroids to maintain hemodynamic stability and analgesia without opioids [8,9].

Studies have shown that dexmedetomidine, approved in December 1999, is the newest alpha₂-adrenoceptor agonist for short-term sedation (<24 hours). These agents provide multiple perioperative benefits, including reduced sympathetic tone, blunted stress responses, lower anesthetic and opioid needs, and calming yet cooperative sedation. They may also help prevent perioperative myocardial ischemia and are increasingly used in pain management and regional anesthesia [11,12].

Originally developed as an intravenous antiarrhythmic, lidocaine has gained attention for its perioperative analgesic and anti-inflammatory properties. Evidence suggests that intravenous lidocaine (IVL) reduces postoperative pain and opioid consumption. Emerging applications include preventing propofol-induced injection pain, reducing hyperalgesia, relaxing bronchial smooth muscle, and enhancing general anesthesia depth. IVL has a favorable pharmacokinetic profile, with rapid distribution, hepatic metabolism, and a high therapeutic index; plasma levels typically remain well below toxic thresholds [12,13].

Other non-opioid options include fascial plane blocks and epidural anesthesia. Studies have shown that in laparoscopic abdominal procedures, epidural ropivacaine provides superior pain control during surgery and better pain scores afterward compared to IV fentanyl, without limiting movement. Also, preemptive epidural analgesia is a potential approach to regulating immune function during surgery and improving postoperative pain control [14-18].

This study evaluated multimodal opioid-free analgesia using intravenous lignocaine, dexmedetomidine, and epidural ropivacaine. The main objectives were to assess intraoperative and postoperative hemodynamic parameters (mean arterial pressure, heart rate, oxygen saturation), to assess intraoperative and postoperative analgesia, and to assess postoperative sedation and cognitive functions after using OFA in laparoscopic surgeries. The goal was to assess its efficacy in providing postoperative pain relief while minimizing adverse outcomes.

## Materials and methods

This study aims to assess the efficacy of OFA for maintaining hemodynamic stability and managing pain in laparoscopic surgeries. Its specific objectives are: (1) to evaluate perioperative hemodynamic parameters (mean arterial pressure (MAP), heart rate (HR), oxygen saturation (SpO2)); (2) to measure intraoperative and postoperative analgesic requirements; (3) to assess postoperative sedation levels and cognitive function; and (4) to determine postoperative outcomes specifically when OFA is combined with epidural analgesia alongside general anesthesia.

We believe OFA offers a daring alternative for laparoscopic surgeries. We are confident it will provide good hemodynamic stability, effective pain control, reduce rescue needs, and lead to clearer-headed patients with faster cognitive recovery postoperatively. Combining it with epidural analgesia is a step, aiming for smoother recovery, fewer complications, and happier patients, proving that laparoscopic surgery can be effectively managed without starting the opioid cascade.

This prospective observational study was conducted at a tertiary care hospital from January 2021 to June 2022 after obtaining approval from the Institutional Ethics Committee (Human Research), C.U. Shah Medical College, Surendranagar (Approval: CUSMC/IEC(HR)/DI/1/2021/FINAL APPROVAL/30/2022). All participants provided written informed consent following a detailed explanation of the study in their preferred language. The study included patients aged 18-65 years classified as American Society of Anesthesiologists (ASA) grade I-III, who were scheduled for laparoscopic surgeries, including total laparoscopic hysterectomy, laparoscopic cholecystectomy, and laparoscopic hernia repair surgery.

Any patient refusal or patient contraindicated for epidural anesthesia (allergy to local anesthetics, bleeding diathesis, infection at injection site, pre-existing neurological deficits), pregnant patients, and patients not fitting the above criteria were excluded. The research was carried out with 50 eligible patients enrolled after thorough pre-anesthetic evaluation.

Preoperative preparation included routine laboratory investigations and placement of a large-bore intravenous cannula for Ringer's lactate infusion. Standard premedication consisting of intravenous ondansetron (0.15 mg/kg), midazolam (0.02 mg/kg), and glycopyrrolate (0.01 mg/kg) was administered 15 minutes prior to induction.

Intraoperative management began with standard monitoring, including pulse oximetry, non-invasive blood pressure measurement, and electrocardiography. Epidural catheterization was performed at the L1-L2 interspace using an 18-G Tuohy needle under strict aseptic conditions, with a test dose of 0.5% ropivacaine (10 mL + 5 mL) administered based on hemodynamic response. Following preoxygenation with 100% oxygen for three minutes via bag-mask ventilation, anesthesia was induced with dexmedetomidine infusion (1 μg/kg over 10 minutes), 2% lignocaine (1.5 mg/kg), propofol (1.5 mg/kg), and succinylcholine (1.5 mg/kg). Endotracheal intubation was confirmed by capnography and bilateral chest auscultation.

Anesthesia maintenance was achieved using isoflurane in oxygen along with atracurium (0.5 mg/kg loading dose, followed by 0.1 mg/kg maintenance doses) for neuromuscular blockade, guided by train-of-four monitoring. Additional dexmedetomidine infusion (0.2-0.3 μg/kg/hr) was administered as needed. Reversal of neuromuscular blockade was accomplished with neostigmine (0.05 mg/kg) and glycopyrrolate (10 μg/kg), with extubation performed upon achieving a train-of-four ratio ≥4, adequate oxygenation on room air, and return of protective airway reflexes.

Hemodynamic parameters, including heart rate, blood pressure, and oxygen saturation, were recorded at baseline, after non-depolarizing neuromuscular blocking agent (NDMR), every 30 minutes intraoperatively for two hours, and hourly for four hours postoperatively. Postoperative analgesia was maintained with 0.2% ropivacaine (10 mL boluses every 12th hour via epidural catheter) for 24 hours, supplemented with intramuscular diclofenac (75 mg) for breakthrough pain (visual analog scale (VAS) >5) as rescue analgesia. Pain scores were assessed using the VAS at extubation and subsequently at 0.5, 1, 2, 3, 4, 6, 12, 18, and 24 hours postoperatively. VAS scores were calculated as follows: score 0-1: no pain; score 2-3: mild pain; score 4-6: moderate pain; score 7-8: severe pain; score 9-10: very severe pain.

Sedation levels were evaluated using the Ramsay Sedation Scale (RSS) immediately after extubation and at 30-minute intervals for the first four hours.

The RSS is scored as follows: level response - (1) awake, anxious, agitated, restless; (2) awake, cooperative, oriented, tranquil; (3) awake, responds only to commands; (4) asleep, brisk response to light glabellar tap or loud auditory stimulus; (5) asleep, sluggish response to light glabellar tap or loud auditory stimulus; (6) no response to light glabellar tap or loud auditory stimulus.

Cognitive function was assessed using the Montreal Cognitive Assessment (MoCA), performed 24 hours before and after surgery.

The MoCA is a widely used, brief screening tool designed to detect mild cognitive impairment. It assesses various cognitive domains, including visuospatial/executive function, naming, memory, attention, language, abstraction, delayed recall, and orientation. The MoCA is a 30-point test typically completed in about 10 minutes and has been validated in multiple countries.

Postoperative complications, including nausea, vomiting, pruritus, urinary retention, respiratory depression, and disorientation, were monitored for 24 hours.

Statistical analysis was performed using SPSS version 22.0 (IBM Corp., Armonk, NY), Microsoft Excel 2019 (Microsoft Corporation, Redmond, WA), and MedCalc version 12.5 (MedCalc Software Ltd, Ostend, Belgium) software packages.

## Results

Demographic data of subjects

The study population comprised 50 participants with a mean age of 41.6 years (95% CI: 38.133 to 45.067). The cohort included eight male participants (mean age: 40.5 years; 95% CI: 28.48 to 52.07) and 42 female participants (mean age: 40.84 years; 95% CI: 37.274 to 44.406). The complete demographic distribution of the study population is presented in Table 1.

**Table 1:** Demographic data of all patients.

Age group	Gender		Total	Percentage
Years	Male	Female		
11-20	1	0	1	2%
21-30	1	9	10	20%
31-40	2	12	14	28%
41-50	2	13	15	30%
51-60	1	6	7	14%
61-70	1	1	2	4%
71-80	0	1	1	2%
Total	8	42	50	100%

Mean of various parameters

Evaluation of mean hemodynamic values revealed maintained stability during surgical intervention and recovery phases, as shown in Table 2.

**Table 2:** Mean of various parameters. HR: heart rate; SBP: systolic blood pressure; DBP: diastolic blood pressure; SpO2: oxygen saturation; NDMR: non-depolarizing neuromuscular blocking agent.

Parameter	Baseline	After intubation	After NDMR	15 min	30 min	45 min	1 hour	1.15 hours	1.3 hours	1.45 hours	2 hours	At extubation	Postop 1 hour	2 hours	3 hours	4 hours
																	HR (/min)	86.78	86.38	82.36	82.6	83.18	83.54	84	85	88	85	85	85	85	85	86	86
SBP (mmHg)	128	123	111	110	110	114	115	114	110	111	118	117	118	118	118	117
DBP (mmHg)	80	77	72	71	69	71	76	76	73	74	76	76	77	76	76	74
SpO2 (%)	99.34	100	100	100	100	100	100	100	100	100	100	99.72	99.06	99.26	99.14	99.02

The mean of heart rate demonstrated stable maintenance across both intraoperative and postoperative periods, with no clinically significant fluctuations. Baseline heart rate was 86.8 ± 10.57 beats per minute (bpm), showing moderate variability. After intubation, it slightly decreased to 86.4 ± 6.92 bpm, with reduced variability. Post-NDMR heart rate was (82.4 ± 6.38 bpm), stabilizing near 82-84 bpm over the next hour. A transient peak of 88 ± 8 bpm was observed at 1.30 hours, before returning to ~85 bpm by extubation. Postoperatively, heart rate remained stable in the mid-85s (±6-6.14 bpm) over four hours, indicating consistent recovery. Standard deviations narrowed post-baseline, suggesting less inter-individual variability.

The systolic blood pressure (SBP) remained stable throughout. Baseline SBP was 127.5 ± 9.12 mmHg, decreasing post intubation (122.8 ± 8.32 mmHg) and dropping further after NDMR (111.4 ± 5.15 mmHg). Pressures remained lower during the first 1.5 hours (lowest at 109.7 ± 3.37 mmHg at 1.30 hours) before gradually recovering. By two hours, values rose to 117.8 ± 8.77 mmHg, stabilizing post extubation (~117-118 ± 5.5-7.45 mmHg) over the next four hours. Variability (SD) was highest at baseline and two hours, while narrower deviations during mid-procedure (e.g., 3.37-5.11 mmHg) suggest tighter hemodynamic control. Postoperative pressures remained near pre-extubation levels, indicating sustained recovery.

The diastolic blood pressure (DBP) of all patients showed minor variations. There is a slight decrease in blood pressure after 15 minutes of NDMR introduction. The DBP remained stable throughout. Baseline DBP was 80.3 ± 5.35 mmHg, declining after intubation (76.9 ± 5.13 mmHg) and further post NDMR (72.2 ± 4.08 mmHg), reaching its lowest point at 30 minutes (68.6 ± 3.11 mmHg). After one hour, DBP was 76.3 ± 4.77 mmHg, with fluctuations between 72 and 76 mmHg over subsequent intervals. Post-extubation and postoperative pressures stabilized near baseline levels (~74-76.6 mmHg), with variability (SD) lowest during mid-procedure (e.g., 3.11-4.08 mmHg) and higher at baseline, two hours post operation (6.66 mmHg), and extubation (5.64 mmHg). Postoperative values showed gradual stabilization, ending at 74.2 ± 3.42 mmHg by four hours.

The mean of O2 saturation of all patients showed no change in O2 saturation throughout the perioperative period in any patients.

**Figure 1:**
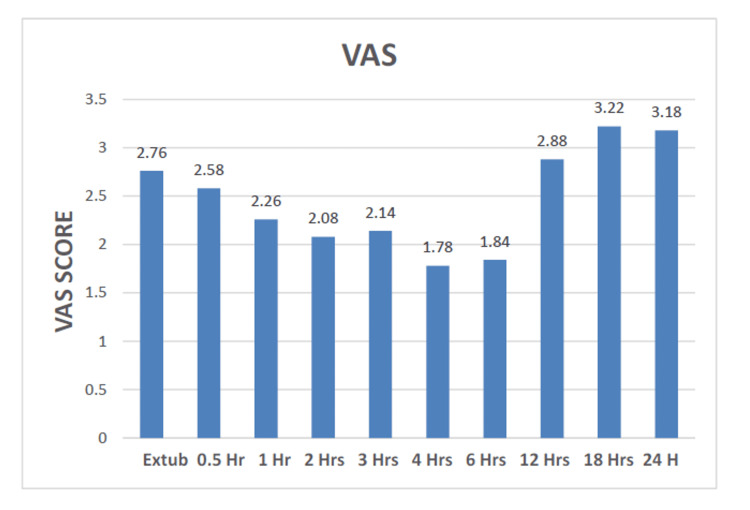
Mean of visual analog scale (VAS).

Table 3 and Figure 1 show the mean VAS of all patients. The score suggests mild pain in the initial postoperative period, which decreases gradually four to five hours postoperatively. Mild pain was seen after 12 hours postoperatively. A VAS of more than 5 was never achieved in any patient.

**Table 3:** Visual analog scale (VAS).

VAS score	Number	Mean	SD
At extubation	50	2.76	0.64
0.5 hours	50	2.58	0.72
1 hour	50	2.26	0.83
2 hours	50	2.08	0.83
3 hours	50	2.14	0.55
4 hours	50	1.78	0.54
6 hours	50	1.84	0.71
12 hours	50	2.88	0.75
8 hours	50	3.22	0.76
24 hours	50	3.18	0.7

The RSS data for 50 patients show a progressive decline in sedation levels post extubation. At extubation, the mean RSS was 3.72 ± 0.48, indicating moderate sedation. Scores steadily decreased over time, 2.88 ± 0.50 at 0.5 hours, 2.46 ± 0.42 at one hour, and 2.1 ± 0.47 at two hours, reflecting a gradual return to wakefulness. Sedation reached its lowest mean at three hours (1.94 ± 0.20), with minimal variability, suggesting consistent light sedation. However, at four hours, the mean slightly increased to 1.96 alongside a notable spike in variability (SD: 1.13), indicating divergent recovery patterns among patients. Overall, sedation diminished predictably in the first three hours.

The MoCA score of patients 24 hours after extubation was almost similar to that one day before surgery.

Out of 50 patients, only nine (18%) developed complications like nausea and vomiting, and 41 (82%) patients had no complications.

## Discussion

Our observational study investigated the impact of OFA on perioperative stress response, demonstrating improved hemodynamic stability that may enhance patient safety during general anesthesia.

Heart rate variability

The mean baseline heart rate was 86.78 ± 10.57 bpm, which decreased to 82.36 ± 6.916 bpm post induction. Intraoperative measurements revealed stable values at 84 ± 6 bpm (one hour), 85 ± 6 bpm (two hours), and 86 ± 6.07 bpm (four hours postoperative), confirming consistent hemodynamic maintenance throughout all surgical phases. These findings align with previous studies, including Panchgar et al. [19], who reported attenuated hemodynamic stress responses with dexmedetomidine, noting only two cases of bradycardia attributed to controlled infusion rates. Similarly, Swaika et al. documented statistically significant intergroup heart rate differences at four hours (p < 0.05) and 24 hours postoperatively. The observed stability (mean variation range: 82.36-86.78 bpm) suggests effective stress response modulation, a favorable safety profile (4% bradycardia incidence in our cohort), and protocol reproducibility with the slow bolus infusion technique. These results support the reliability of the approach in maintaining hemodynamic stability during surgery [20].

Variation in blood pressure

Our study demonstrated consistent blood pressure maintenance during major laparoscopic procedures under OFA. These findings correlate with prior research, including Bakan et al. [12], who reported statistically significant hemodynamic variations, noting increased hypotensive events in group RF (or opioid-based anesthesia with remifentanil and propofol infusions), and more hypertensive episodes in group DL (dexmedetomidine, lidocaine, and propofol infusions). Additionally, Swaika et al. [20] and Panchgar et al. [19] demonstrated superior blood pressure control with dexmedetomidine, highlighting enhanced stability in SBP, DBP, and MAP with a significant advantage over control groups (p < 0.05). Clinically, the observed pressure range (SBP: 110-118 mmHg; DBP: 69-76 mmHg) indicates less than 10% variation from baseline values, no clinically significant hypotension (all values remained >20% above critical thresholds), and a stable intraoperative-to-postoperative transition. These results confirm the efficacy of OFA in maintaining hemodynamic stability while minimizing adverse fluctuations, supporting its use as a reliable alternative in major laparoscopic surgeries.

Postoperative analgesia

Visual Analog Scale Score

Pain assessment using the VAS revealed consistent and acceptable pain relief (VAS ≤ 3) throughout the postoperative period in our study. The recorded scores were as follows: extubation (2.76 ± 0.64), 0.5 hours (2.58 ± 0.72), one hour (2.26 ± 0.83), two hours (2.08 ± 0.83), three hours (2.14 ± 0.55), four hours (1.78 ± 0.54), six hours (1.84 ± 0.71), 12 hours (2.88 ± 0.75), 18 hours (3.22 ± 0.76), and 24 hours (3.18 ± 0.7). These findings align with existing literature, including Toleska et al. [21], who reported complete pain absence (VAS 0) at 24 hours in OFA patients, even during coughing. Similarly, Bakan et al. [12] observed significantly reduced pain scores (p < 0.05) and lower rescue analgesic requirements in OFA groups. Ahmed et al. [22] further demonstrated OFA benefits, including prolonged time to first rescue analgesia (54.14-99.11 minutes, p < 0.0001), reduced pain scores at four/six hours (p < 0.05), and decreased morphine consumption (0.505-2.396 mg, p = 0.003). Choi et al. [23] also reported superior pain control, with lower 30-minute postoperative VAS (4.1 ± 2.1 vs. 6.3 ± 2.3, p < 0.001) and reduced post-anesthesia care unit (PACU) rescue analgesia needs (21.6% vs. 73.7%, p < 0.001). Clinically, our results confirm effective analgesia, with all VAS scores remaining ≤3.22/10, peak pain relief at four hours (1.78 ± 0.54), and late-phase scores (18-24 hours) staying near the acceptable threshold (3.18-3.22). These findings reinforce the efficacy of opioid-free analgesia, demonstrating outcomes comparable to established benchmarks in the literature.

Ramsay Sedation Scale Score

In the present study, the RSS scores demonstrated significant sedation at extubation (3.72 ± 0.48), followed by a progressive decline at 0.5 hours (2.88 ± 0.50), one hour (2.46 ± 0.42), two hours (2.10 ± 0.47), three hours (1.94 ± 0.20), and four hours post extubation (1.96 ± 1.13). These findings correlate with Swaika et al. [20], who reported statistically higher RSS scores in dexmedetomidine groups at four to 24 hours (p < 0.006) while maintaining cooperative sedation. Sharma et al. [24] further supported these results, confirming an increased prevalence of optimal conscious sedation (RSS 2-3) with dexmedetomidine, along with improved patient responsiveness compared to conventional regimens. Additionally, Bakri et al. [25] documented prolonged yet clinically appropriate sedation, with a mean RSS of 4.2 ± 0.8 at six hours of PACU stay, facilitating early recovery. Clinically, the initial RSS of 3.72 indicates effective therapeutic sedation upon emergence, while the steady decline to 1.96 by the fourth hour reflects rapid recovery. The narrowing standard deviation (from ±0.48 at extubation to ±0.20 at three hours) suggests a predictable awakening pattern, though the late-phase variability (±1.13 at four hours) highlights individual recovery differences. These findings reinforce dexmedetomidine’s role in ensuring balanced sedation with favorable recovery dynamics.

Montreal Cognitive Assessment

Among available neurocognitive assessment tools, the MoCA demonstrated optimal sensitivity for detecting postoperative cognitive changes. Our study revealed preoperative baseline MoCA scores of 27.9 ± 1.37, with a modest decline to 26.72 ± 1.37 at 24 hours post extubation (ΔMoCA: -1.18). These findings align with Hakim et al. [26], who reported superior recovery in opioid-free total intravenous anesthesia (OF-TIVA) groups, evidenced by higher 24-hour Quality of Recovery-40 (QOR-40) scores (182.0 (164.0-192.0) vs. 170.0 (156.0-185.0) in opioid groups; p = 0.03). Similarly, Song et al. [27] observed significantly enhanced recovery in OFA patients, with QoR-40 scores on postoperative day one measuring 166.9 ± 17.8 (OFA) versus 155.9 ± 21.2 (regional anesthesia), yielding a mean difference of -11.0 (95% CI: -20.0, -2.0; p = 0.018). Clinically, the minimal MoCA score reduction (-1.18 points) suggests neuroprotective benefits of OFA, corroborating established QoR-40 improvements in OFA literature. These results underscore MoCA's utility as a sensitive tool for postoperative cognitive monitoring, capable of detecting subtle yet clinically relevant neurocognitive changes.

Postoperative Nausea and Vomiting

In our study cohort, 18% of patients experienced postoperative nausea and vomiting (PONV), which was effectively managed with intravenous ondansetron (0.15 mg/kg). This incidence rate demonstrates the favorable antiemetic profile of our OFA protocol. Comparative literature strongly supports these findings. Ahmed et al. [22] reported a significant reduction in PONV impact scale with OFA (p = 0.022), while Chen et al. [28] documented lower PONV prevalence in the OFA group (26.3%) compared to controls (68.4%), with an odds ratio of 0.31 (95% CI: 0.158-0.589, p < 0.001) and reduced antiemetic use (5.3% vs. 28.9%). Additionally, Song et al. [27] found significantly lower nausea incidence (p = 0.014) and reduced shivering frequency (p = 0.025) in OFA groups. Clinically, our observed PONV rate of 18% compares favorably with conventional opioid-based anesthesia (typically 30-80%) and aligns with literature-reported OFA outcomes (26.3%) in Chen et al. [28]. The 0.15 mg/kg ondansetron dose proved effective for rescue treatment, further supporting OFA as a strategic approach for PONV reduction in surgical patients.

## Conclusions

This observational study concludes that OFA, combining epidural ropivacaine with dexmedetomidine-propofol general anesthesia, is a safe and effective strategy for maintaining perioperative hemodynamic stability and postoperative analgesia in major laparoscopic surgeries. The technique demonstrated consistent hemodynamics, with no statistically significant variations in heart rate, systolic/diastolic blood pressure, or mean arterial pressure at critical intervals, including after neuromuscular blockade reversal and up to four hours postoperatively. Postoperative pain control was effective, as evidenced by mild pain at extubation (VAS scores) that steadily decreased over 30 minutes to 24 hours, supported by scheduled epidural ropivacaine. While RSS scores were at peak at extubation, they normalized within four hours, suggesting transient sedation without prolonged effects. Cognitive function remained intact (no significant MoCA score changes). Postoperative nausea and vomiting were the only notable complications, with no other adverse events reported. These findings highlight the viability of OFA as a promising alternative for reducing opioid reliance while ensuring stable intraoperative conditions, adequate analgesia, and favorable recovery outcomes. Further randomized controlled trials with larger cohorts are recommended to validate these results and refine protocols.
